# Preimplantation genetic testing for X-linked chronic granulomatous disease induced by a *CYBB* gene variant: A case report

**DOI:** 10.1097/MD.0000000000037198

**Published:** 2023-02-02

**Authors:** Xinlian Chen, Cuiting Peng, Han Chen, Fan Zhou, Yuezhi Keqie, Yutong Li, Shanling Liu, Jun Ren

**Affiliations:** a Department of Medical Genetics, Center for Prenatal Diagnosis, West China Second University Hospital, Sichuan University, Sichuan, China; b Department of Obstetrics and Gynecology, West China Second University Hospital, Sichuan University, Sichuan, China; c Key Laboratory of Birth Defects and Related Diseases of Women and Children (Sichuan University), Ministry of Education, Sichuan, China.

**Keywords:** *CYBB* gene, haplotyping, PGT-M, X-linked chronic granulomatous disease

## Abstract

**Introduction:**

X-linked recessive chronic granulomatous disease (XR-CGD) is a severe primary immunodeficiency principally caused by a *CYBB* (OMIM: 300481) gene variant. Recurrent fatal bacterial or fungal infections are the main clinical manifestations of XR-CGD.

**Patient concerns:**

In the current case, in vitro fertilization (IVF) associated with preimplantation genetic testing for monogenic disorder (PGT-M) was applied for a Chinese couple who had given birth to a boy with XR-CGD.

**Diagnosis:**

Next-generation sequencing-based SNP haplotyping and Sanger-sequencing were used to detect the *CYBB* gene variant (c.804 + 2T>C, splicing) in this family.

**Interventions:**

The patient was treated with IVF and PGT-M successively.

**Outcomes:**

In this IVF cycle, 7 embryos were obtained, and 2 of them were euploid and lacked the *CYBB* gene variant (c.804 + 2T>C). The PGT results were verified by prenatal diagnosis after successful pregnancy, and a healthy girl was eventually born.

**Conclusion:**

PGT-M is an effective method for helping families with these fatal and rare inherited diseases to have healthy offspring. It can availably block the transmission of disease-causing loci to descendant.

## 1. Introduction

Chronic granulomatous disease (CGD) is an infrequent and inherited primary immunodeficiency with significant mortality.^[1]^ It is caused by functional impairment of the nicotinamide adenine dinucleotide phosphate (NADPH) oxidase complex in neutrophilic granulocytes and monocytes.^[2]^ CGD is characterized by recurrent and severe infections, dysregulated inflammation, and autoimmunity^[2]^ because phagocytic cells cannot produce enough hydrogen peroxide to kill catalase-positive bacteria and fungi. In the United States^[3]^ and Europe,^[4]^ the incidence of CGD ranges from approximately 1/200,000 to 1/250,000 live births. To date, the incidence of CGD has not been reported in China.

In terms of the molecular genetic aspect of CGD, we can divide this disease into X-linked recessive CGD (XR-CGD, OMIM: 306400) and autosomal recessive CGD (AR-CGD). XR-CGD is caused by variations in the *CYBB* gene, which encodes the gp91^phox^ protein, while AR-CGD is due to variants of *CYBA, NCF1, NCF2*, or *NCF4*, which encode p22^phox^, p47^phox^, p67^phox^, and p40^phox^, respectively.^[5]^

The primary clinical presentation of patients with CGD is infection early in life. Patients with X-linked CGD usually develop disease earlier and have more severe symptoms than those with AR-CGD.^[6,7]^ The most common types of infection in CGD patients are pneumonia, lymphadenitis, and abscess. Patients are more susceptible to catalase-positive organisms. Invasive fungal infection is still a common cause of death in patients with CGD.^[6,7]^ Another clinical presentation of patients with CGD is inflammation and autoimmunity, such as inflammatory bowel disease (IBD). CGD is mainly treated with antibiotics and antifungal drugs to prevent and cure infections. Other therapies include injection of interferon-γ, granulocyte infusion, and hematopoietic stem cell transplantation (HSCT). Currently, HSCT is the only treatment that can cure CGD, but it is very difficult to find an HLA-matched donor. The long-term efficacy and safety of gene therapy for CGD need to be further studied. To date, no gene therapy drugs have been successfully applied for the clinical treatment of CGD.

Preimplantation genetic testing for monogenic disorder (PGT-M) is an effective method for helping families with these fatal and rare inherited diseases to have healthy offspring. The PGT-M technique can select unaffected embryos to block the transmission of disease-causing variants to offspring. In addition, for diseases such as congenital immune deficiency disease and certain lymphomas, which can be cured by HSCT, PGT-M can also be used to select embryos for which HLA typing is in line with ailing siblings. Thus, cord blood stem cells from the child born by PGT-M and HLA typing could be used to treat the ailing sibling.

In this case, we reported a female who is a *CYBB* gene variant (c.804 + 2T>C, splicing) carrier through PGT-M to have a healthy infant. Sanger-sequencing and next-generation sequencing (NGS)-based haplotyping were used for PGT-M. The normal embryos were used for preimplantation genetic testing for aneuploidy (PGT-A) by NGS. Eventually, an embryo with a score of 4BC was transplanted and impregnated successfully. The result of prenatal diagnosis was consistent with PGT-M, resulting in the birth of a healthy girl.

## 2. Materials and methods

### 2.1. Patients

The proband boy died of X-linked chronic granulomatous disease at the age of 3 years. The child was admitted to West China Second University Hospital at the age of 1 year old because of severe pneumonia, sepsis, abnormal liver function, severe anemia, and congenital immune deficiency.

Whole-exon sequencing results showed that the proband had a hemizygous variant in the *CYBB* (OMIM:300481, NM_000397) gene c.804 + 2T>C, splicing, X-linked recessive inheritance. According to the standards and guidelines for sequence variant interpretation of the American College of Medical Genetics and Genomics (ACMG/AMP),^[8]^ the pathogenicity of the variant was assessed as likely pathogenic, PVS I + PM2_Supporting. This variant is mainly associated with X-linked chronic granulomatous disease (CGDX, OMIM:306400). The variant is inherited from the proband’s mother, the father is normal at this locus.

### 2.2. In vitro fertilization (IVF) and trophectoderm biopsy

Controlled ovarian stimulation, intracytoplasmic sperm injection (ICSI), trophectoderm biopsy and embryo transfer were conducted in the Reproductive Medicine Center of West China Second University Hospital (Sichuan University) according to the standard protocol.^[9,10]^

In this IVF cycle, 7 embryos developed into blastocysts, and the trophectoderm cells were biopsied on day 5 or 6 after insemination by ICSI. Approximately 5 to 8 biopsied cells were transferred into 4.5 μL lysis buffer (Yikon Genomics) in polymerase chain reaction (PCR) tubes for whole-genome amplification (WGA).

### 2.3. gDNA extraction and whole genome amplification

gDNA was extracted from peripheral blood lymphocytes (fresh blood samples collected in EDTA-stabilized anticoagulative tubes) using the DNeasy Blood and Tissue Kit (Qiagen). The multiple annealing and looping-based amplification cycle (MALBAC, Yikon genomics) method was performed for WGA.

### 2.4. Variant detection

PCR amplification and Sanger-sequencing were conducted to validate the *CYBB* variant. Primers were designed to amplify the segment including c.804 + 2T>C variant of the *CYBB* gene.

Forward primer (GCCTACATCAGAGCACTTA) and reverse primer (GCTGTTCCATGACATTTG T) were designed (by Primer 5.0 software) and synthesized (by TsingKe Biotechnology). PCR was performed in a 25-μL system using 2×GoldStar Best Master Mix (CWBIO) on a 96-Well Thermal Cycler Veriti Dx (Life Technologies).

The amplification system contained 2 μL of primer mix, 12.5 μL of enzyme mix, 1 μL gDNA or purified WGA products as template. The reaction conditions were as follows: 95 °C for 10 minutes; 94 °C for 30 seconds, 64 °C for 30 seconds, 72 °C for 45 seconds (3 cycles); 94 °C for 30 seconds, 62 °C for 30 seconds, 72 °C for 45 seconds (5 cycles); 94 °C for 30 seconds, 60 °C for 30 seconds, 72 °C for 45 seconds (5 cycles); 94 °C for 30 seconds, 58 °C for 30 seconds, 72 °C for 45 seconds (25 cycles); 72 °C for 5 minutes; held at 4 °C. Sanger sequencing was performed by TsingKe Biotechnology, and the data were analyzed by ChromasPro software.

### 2.5. Library preparation and NGS

Purified WGA products and gDNA were used for SNP and CNV library preparation by an NGS library preparation kit (Yikon Genomics). All operations followed the manufacturers’ instructions. The SNP library was sequenced by the MiSeq Dx platform (Illumina), and the CNV library was sequenced by the NextSeq CN500 platform (Illumina). The raw MiSeq data were automatically filtered, and FASTQ files were generated. The raw NextSeq CN500 data were filtered, and FASTQ files were generated by ChromGo software (Yikon Genomics).

### 2.6. NGS-based SNP haplotyping and CNV analysis

To avoid misdiagnosis caused by allele drop-out (ADO) and homologous recombination of chromosomes, NGS-based haplotyping was conducted using SNPs within the 2-million base pair (Mb) region flanking the targeted gene. A total of 131 SNPs were selected in this case. For X-linked recessive hereditary diseases such as X-CGD, the informative SNP sites are homozygous in the male and proband, heterozygous in the female carrier. ChromGo software (Yikon Genomics) was used for bioinformatics analysis.

### 2.7. Embryo transplantation and prenatal diagnosis

For embryo transfer recommendations, we organized the Reproductive Medicine-Medical Genetic Multidisciplinary Treatment, which involves clinical geneticists, reproductive doctors and embryologists. The priority of embryo transfer was based on the results of PGT M&A and morphological scores. Prenatal diagnosis was applied for amniotic fluid cells at gestational weeks 18–22^+6^ by chromosomal microarray (cytoscan 750K, Affymetrix) and Sanger-sequencing. The results of prenatal diagnosis were consistent with PGT. Eventually, the woman delivered a healthy baby girl.

## 3. Results

### 3.1. Patients and genetic background

The couple had a boy who died at 3 years old. When he was 1 year old, the proband presented with fever and was diagnosed with sepsis, lung infection, abnormal liver function, and moderate anemia (iron deficiency, infection). Pathological diagnosis of the left axillary lymph nodes suggested granulomatous inflammation with necrosis. The patient was eventually diagnosed with CGD. When he was 2 years old, the proband received hematopoietic stem cell transplantation, but unfortunately graft-versus-host disease occurred.

**Figure 1.**
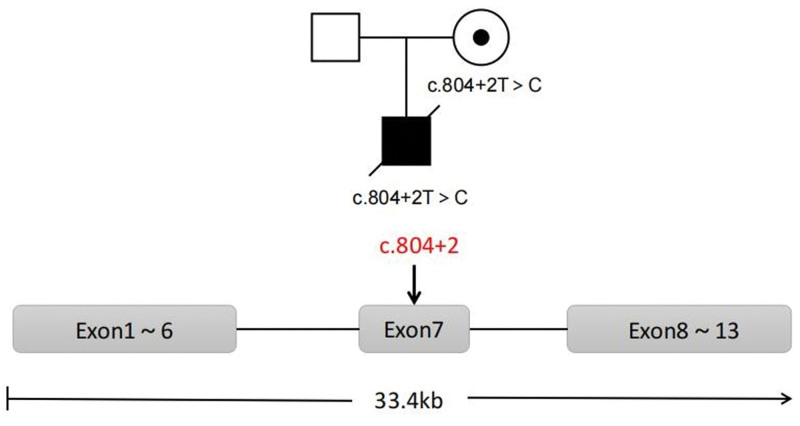
Genogram of the family. The female is a carrier of the *CYBB* gene c.804 + 2T>C, the male is normal, and the proband is a deceased hemizygous patient. The *CYBB* gene with 13 exons is located in Xp11.4. The variant c.804 + 2T>C is located at exon 7.

After the boy died, the couple visited the Department of Medical Genetics/Center of Prenatal Diagnosis, West China Second University Hospital (Sichuan University) for genetic counseling, hoping to have another healthy child through the PGT-M. Whole-exon sequencing was applied in this family, and the results showed that the proband had a variant in the *CYBB* gene (c.804 + 2T>C, splicing), and he was a hemizygote. The female also had a variant in the *CYBB* gene (c.804 + 2T>C, splicing), and she is a carrier. No abnormality was found in the above gene locus in the husband. The family tree is shown in Figure 1.

The *CYBB* gene contains 33.4 kb and 13 exons (Fig. 1). The variant c.804 + 2 is located at exon 7, ChrXp11.4. According to the ACMG/AMP guidelines, we evaluated this variant as PVS1+PM2_Supporting, Likely Pathogenic. This variant is associated with XR-CGD.

### 3.2. Detection of variant by PCR and Sanger-sequencing

**Figure 2.**
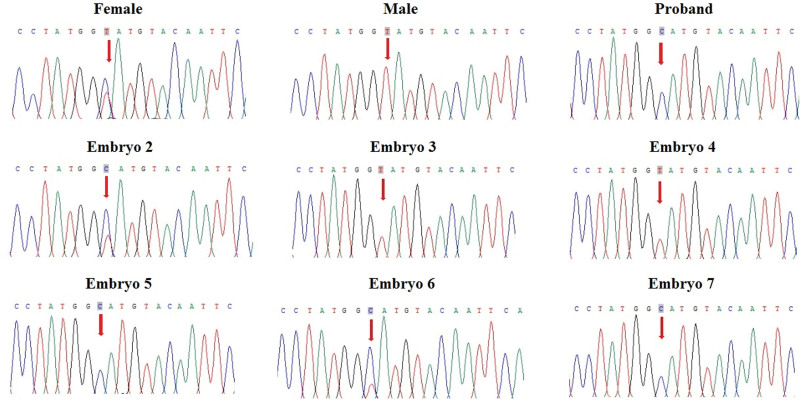
Results of Sanger sequencing for the variant. The figure shows that females E02 and E06 are carriers of the *CYBB* gene c.804 + 2 T>C variant, the proband, E05 and E07 are hemizygotes, and no variants of the *CYBB* gene c.804 + 2 T>C were detected in males E03 and E04. For X-linked recessive inherited diseases, we recommend that the embryo sex be determined to facilitate the analysis. PCR amplification of *SRY* gene-specific fragments and agarose gel electrophoresis are simple and effective methods for embryo sex determination (data not shown).

gDNA was extracted from peripheral blood lymphocytes of this couple. In this PGT cycle, 7 embryos finally developed into blastocysts after ICSI. Biopsied trophectoderm cells were amplified by multiple annealing and looping-based amplification cycles. Polymerase chain reaction (PCR) amplification and Sanger sequencing were conducted to detect the *CYBB* gene variant. Primers were designed to amplify the segment including c.804 + 2 T>C of the *CYBB* gene. The results of Sanger sequencing show that the female is a carrier of the *CYBB* gene c.804 + 2 T>C variant, and the proband is hemizygote (Fig. 2). For embryos, E02, E05, E06 and E07 were detected as c.804 + 2 T>C of the *CYBB* gene, and E03 and E04 were normal in this locus (Fig. 2). E01 failed Sanger sequencing (Fig. 2). For sex-linked inherited diseases, we performed a sex-identification test for embryos by PCR amplifying the *SRY* gene-specific sequences on the Y chromosome (data not shown). The results of Sanger sequencing of the variant and embryo sex were considered overall: E02 and E06 are heterozygous females, and E05 and E07 are hemizygote males (patient males). E03 and E04 are normal males and females, respectively.

### 3.3. SNP-based haplotyping

**Figure 3.**
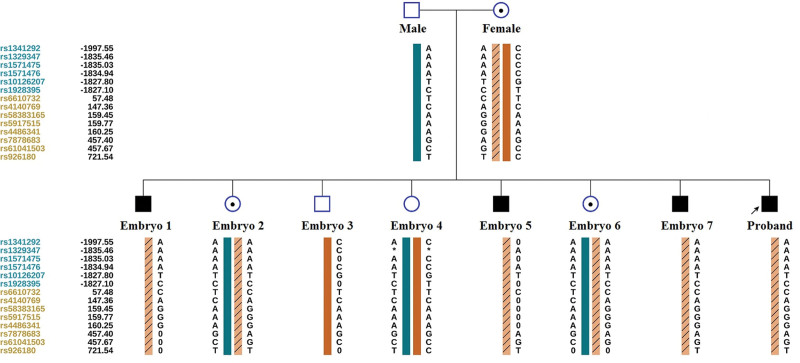
SNP-based haplotyping. The reference SNP cluster ID numbers are listed on the left side. The dark cyan and mustard of ID numbers refer to the downstream and upstream informative SNPs, respectively. The dark cyan bar represents the paternal normal haplotype, the chocolate bar represents the maternal normal haplotype, and the peachpuff bar with a diagonal stripe represents the maternal mutational haplotype. The results show that E01, E05 and E07 are male patients; E02 and E06 are female carriers; E03 is a normal male embryo; and E04 is a normal female embryo.

When the whole genome amplification product is applied for Sanger-sequencing, problems such as sequencing failure, allele drop-out and chromosome recombination may occur. These problems can lead to failed diagnostics or even misdiagnosis. Therefore, we currently diagnose embryos mainly by haplotyping analysis combined with variant detection. SNPs within the upstream and downstream 2 Mb regions flanking the target gene were applied for haplotyping. For the *CYBB* gene, 131 SNPs were chosen for haplotyping. The haplotyping and selection of informative SNPs were performed according to the ESHRE PGT Consortium good practice recommendations for detecting monogenic disorders.^[11]^ In the haplotype diagram (Fig. 3), the dark cyan bar represents the paternal normal haplotype, the chocolate bar represents the maternal normal haplotype, and the peachpuff bar with a diagonal stripe represents the maternal mutational haplotype. Hence, it is clear that E01, E05 and E07 are male patients (c.804 + 2 T>C of *CYBB* gene, hemizygote), and E02 and E06 are female carriers (c.804 + 2 T>C of *CYBB* gene, heterozygote). E03 and E04 are normal males and females, respectively.

### 3.4. Preimplantation genetic testing for aneuploidy

**Figure 4.**
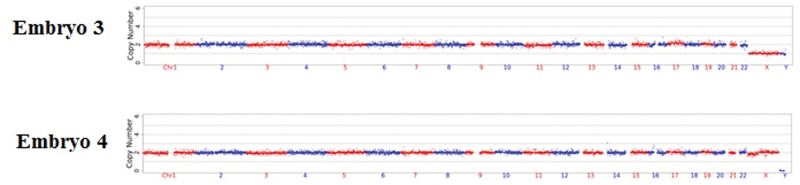
PGT-A results of normal embryos. The results show that no CNVs larger than 10 M and aneuploidy were found in E03 and E04.

After genetic counseling and informed consent, this couple decided to perform PGT-A only in embryos that did not carry the *CYBB* gene c.804 + 2 T>C variant. Therefore, PGT-A was applied for E03 and E04. The results of PGT-A showed that there was no aneuploidy, and copy number variation (CNV) larger than 10 M was found in E03 and E04 (Fig. 4). In the NGS-based PGT-A platform, we only reported CNVs larger than 10 Mb and 30% to 70% mosaicism (larger than 30 Mb).

### 3.5. Embryo transplantation and prenatal diagnosis

In the Reproductive Medicine-Medical Genetic Multidisciplinary Treatment of the West China Second University Hospital, the couple had discussions with reproductive doctors, embryologists and clinical geneticists before the embryo transplantation decision was made. The decision was made according to the results of PGT-M, PGT-A and the morphology^[12]^ of the embryo (Table 1). We first transplanted embryo E03 into the female’s uterus, and unfortunately, the pregnancy was not successful. E04 had a successful pregnancy on the second transplantation.

**Table 1:** Summary of detection results.

Blastocysts number	Gardner grade	CNV	SNP haplotype	Sanger sequencing
E01	4BB	/	male, patient	c.804 + 2 T>C, hemizygote
E02	4BB-	/	female, carrier	c.804 + 2 T>C, heterozygote
E03	4BC	46,XY	normal male	normal
E04	4BC	46,XX	normal female	normal
E05	4BC	/	male, patient	c.804 + 2 T>C, hemizygote
E06	6BC	/	female, carrier	c.804 + 2 T>C, heterozygote
E07	6BC	/	male, patient	c.804 + 2 T>C, hemizygote

CNV = copy number variation, SNP = single nucleotide polymorphism.

During the second trimester, amniotic fluid cells were obtained by amniocentesis for prenatal diagnosis. Amniotic cells were used for chromosome microarray analysis^[13]^ and Sanger-sequencing (data not shown).

## 4. Discussion

In this case, we reported a couple that included an XR-CGD carrier who gave birth to a healthy girl via IVF and PGT-M. NGS-based SNP haplotyping, Sanger-sequencing and NGS-based PGT-A were used. XR-CGD, which is caused by a *CYBB* gene variant, is a congenital and hereditary immunodeficiency. Recurrent bacterial and fungal infections are the main clinical presentations of this disease. Inflammatory complications, such as IBD, are gradually being recognized as a major cause of morbidity and are still challenging to treat.^[14]^ In addition to symptomatic treatment with antibiotics, antifungal drugs or immunological agents such as interferon γ, gene therapy and hematopoietic stem cell transplantation are the most effective therapies for XR-CGD. However, no gene therapy drugs aimed at XR-CGD have been approved for clinical treatment.

The PGT-M technique is helpful for healthy offspring in couples with disease-causing variants. In addition, PGT for HLA typing is useful for families with siblings who need to undergo HSCT therapy, such as those with severe β-thalassemia, Fanconi anemia, CGD, and severe combined immunodeficiency. The aim of PGT-M-HLA is to establish a pregnancy in which the fetus is HLA compatible with a proband in need of HSCT therapy.^[15]^ The possibility of finding an HLA-matched donor within the family or matching an unrelated donor from the Marrow Donor Program is relatively small.^[15]^ Martine De Rycke and her colleague reported a theoretical opportunity of 18.8% for autosomal or X-linked recessive disorders and of 12.5% for autosomal dominant disorders when HLA typing is performed with testing for PGT-M.^[15]^ In China, the team leader, professor Yanwen Xu, reported PGT-M for β-thalassemia combined with HLA matching by Karyomapping.^[16]^ Their results show that the rates of normal and carriers of β-thalassemia with matched HLA were 15.72% and 14.07%, respectively.^[16]^ Thus, PGT-M-HLA is a promising and effective technique for these families.

In our study, MALBAC was used for whole genome amplification. For most point mutations, MALBAC achieved higher amplification coverage and a lower rate of ADO.^[17]^ MALBAC also performed well in the detection of copy number variation. Sanger-sequencing combined with SNP-based haplotyping was applied for PGT-M. The purpose of this combination is to reduce the risk of misdiagnosis due to ADO and homologous recombination of chromosomes. According to the couple’s request, we performed PGT-A on embryos that did not carry the variant. After a successful pregnancy, prenatal diagnosis was applied for amniotic fluid cells at gestational weeks 18–22^+6^ by chromosomal microarray and Sanger-sequencing. In this IVF cycle, E03 and E04 were diagnosed as normal embryos after PGT-M&A. E04 succeeded in pregnancy on the second transplantation. Eventually, the couple had a healthy baby girl.

In conclusion, PGT has developed into a well-established alternative to invasive prenatal diagnosis, even though genetic testing of few cells is still challenging.^[18]^ Theoretically speaking, PGT-M can be used for any monogenic disorders where the disease-causing locus is clearly identified.^[18]^ At present, PGT has been developed into a safe and effective clinical test. With the progress of genetic testing technology and the gradual improvement of patients’ understanding of genetic diseases, an increasing number of patients with hereditary diseases will benefit from PGT in the future. However, with the development of technology, we still need to pay attention to the ethical issues involved in PGT. In particular, the use of PGT techniques for non-disease-related embryo selection is prohibited.

## Acknowledgments

We are grateful to the family for their cooperation and participation, to the embryology team in the Center of Reproductive Medicine for sample preparation and to Teng Zhang from Yikon Genomics for his kindness work.

## Author contributions

**Conceptualization:** Jun Ren.

**Formal analysis:** Yuezhi Keqie.

**Investigation:** Xinlian Chen, Han Chen.

**Methodology:** Fan Zhou.

**Project administration:** Jun Ren.

**Resources:** Shanling Liu.

**Software:** Yutong Li.

**Supervision:** Shanling Liu.

**Validation:** Cuiting Peng.

**Visualization:** Jun Ren.

**Writing – original draft:** Xinlian Chen.

**Writing – review & editing:** Jun Ren.
